# Challenges and successes in the implementation of option B+ to prevent mother-to-child transmission of HIV in southern Swaziland

**DOI:** 10.1186/s12889-018-5258-3

**Published:** 2018-03-20

**Authors:** David Etoori, Bernhard Kerschberger, Nelly Staderini, Mpumelelo Ndlangamandla, Bonisile Nhlabatsi, Kiran Jobanputra, Simangele Mthethwa-Hleza, Lucy Anne Parker, Sifiso Sibanda, Edwin Mabhena, Munyaradzi Pasipamire, Serge Mathurin Kabore, Barbara Rusch, Christine Jamet, Iza Ciglenecki, Roger Teck

**Affiliations:** 1 Médecins Sans Frontières, Mbabane, Swaziland; 2 Médecins Sans Frontières, Geneva, Switzerland; 3 Sexual and Reproductive Health Unit, Ministry of Health, Mbabane, Swaziland; 4 Médecins Sans Frontières, London, UK; 5 CIBER Epidemiología y Salud Pública, Universidad Miguel Hernández, Alicante, Spain; 6 Swaziland National AIDS Programme (SNAP), Ministry of Health, Mbabane, Swaziland; 7 South African Medical Unit, Médecins Sans Frontières, Cape Town, South Africa; 8 London School of Hygiene and Tropical Medicine, London, UK

**Keywords:** PMTCT, HIV, Retention, ART initiation, EID

## Abstract

**Background:**

Universal antiretroviral therapy (ART) for all pregnant/ breastfeeding women living with Human Immunodeficiency Virus (HIV), known as Prevention of mother-to child transmission of HIV (PMTCT) Option B+ (PMTCTB+), is being scaled up in most countries in Sub-Saharan Africa. In the transition to PMTCTB+, many countries face challenges with proper implementation of the HIV care cascade. We aimed to describe the feasibility of a PMTCTB+ approach in the public health sector in Swaziland.

**Methods:**

Lifelong ART was offered to a cohort of HIV+ pregnant women aged ≥16 years at the first antenatal care (ANC1) visit in 9 public sector facilities, between 01/2013 and 06/2014. The study enrolment period was divided into 3 phases (early: 01–06/2013, mid: 07–12/2013 and late: 01–06/2014) to account for temporal trends. Kaplan-Meier estimates and Cox proportional-hazards regression models were applied for ART initiation and attrition analyses.

**Results:**

Of 665 HIV+ pregnant women, 496 (74.6%) initiated ART. ART initiation increased in later study enrolment phases (mid: aHR: 1.41; later: aHR: 2.36), and decreased at CD4 ≥ 500 (aHR: 0.69). 52.9% were retained in care at 24 months. Attrition was associated with ANC1 in the third trimester (aHR: 2.37), attending a secondary care facility (aHR: 1.98) and ART initiation during later enrolment phases (mid aHR: 1.48; late aHR: 1.67). Of 373 women eligible, 67.3% received a first VL. 223/251 (88.8%) were virologically suppressed (< 1000 copies/mL). Of 670 infants, 53.6% received an EID test, 320/359 had a test result recorded and of whom 7 (2.2%) were HIV+.

**Conclusions:**

PMTCTB+ was found to be feasible in this setting, with high rates of maternal viral suppression and low transmission to the infant. High treatment attrition, poor follow-up of mother-baby pairs and under-utilisation of VL and EID testing are important programmatic challenges.

## Background

The use of antiretroviral drugs (ARVs) for prevention of HIV transmission is widely accepted as a ‘Treatment as Prevention’ strategy. Mono-therapy with Zidovudine (AZT) and combined antiretroviral therapy (ART) are effective in reducing vertical HIV transmission from mother to child [1–7]. Lifelong ART decreases morbidity and mortality in adults across all CD4 counts [8, 9], protects the foetus from HIV infection from the time of conception in subsequent pregnancies and reduces the risk of transmission to partners [10–12]. In 2013, the WHO recommended the use of lifelong ART in all pregnant and breastfeeding women at the time of HIV diagnosis regardless of CD4 count, known then as prevention of mother to child transmission (PMTCT) option B+ (PMTCTB+) [13].

The HIV prevalence in Swaziland is estimated at 31% among adults (18–49 years old) [14], and annual HIV incidence is high at 2.5% [15]. HIV disproportionately affects women and peaks at 49% in the age-group 25-29 years old [15] and is overall 41.1% among pregnant women [15, 16]. Until 2014, the Swaziland National PMTCT Program applied the WHO PMTCTA approach, whereby women with CD4 < 350 and/or WHO Stage 3/4 disease were eligible for lifelong ART. Women not fulfilling these criteria were offered AZT from 14 weeks of gestation, AZT/3TC at delivery for one week, followed by AZT until the end of the breastfeeding period. The infants received Nevirapine syrup immediately after delivery and for the duration of the breastfeeding period or through 6 weeks if they were not breastfed [17, 18]. In 2012 the reported PMTCTA coverage was > 80% and maternal to child transmission estimated at 2.4% at 6 weeks [19].

In 2013, Médecins Sans Frontières (MSF) and the Swaziland Ministry of Health initiated a PMTCTB+ implementation study in the southern Shiselweni region. The objective was to determine feasibility of PMTCTB+ in the public health sector and compile lessons learned to inform national policy and scale-up. Interim results of ART initiation rates during the early study implementation period have been described in detail elsewhere [20]. Here we present the final outcomes of this study from the perspective of the pregnant women (ART initiation and retention in care (RIC) rates, viral load (VL) testing uptake and suppression) as well as of their exposed infants (early infant diagnosis (EID) uptake, and HIV transmission rates).

## Methods

### Setting

This was a prospective PMTCTB+ implementation cohort study in Nhlangano health zone, southern Swaziland. Predominantly rural, it comprises one secondary and 8 primary care facilities providing HIV diagnosis and ART integrated into maternal and child health care services. Full details of the setting and the study have been described elsewhere [20].

From 28 January 2013 to 30 June 2014 newly diagnosed and previously known HIV+ pregnant women aged ≥16 years were enrolled into the study and offered lifelong ART at the first ante-natal care (ANC) visit. At completion of study enrolment, PMTCTB+ was adopted as the standard of care nationally, and the study follow-up period coincided with the national roll out. Trained HIV testing counsellors (HTCs) performed HIV testing, treatment preparation, and ART follow-up counselling. A serial rapid testing algorithm was applied using the Alere Determine™ HIV-1/2 rapid test followed by the Uni-Gold™ test on whole blood. Trained nurses initiated ART and performed follow-up visits and ART refills, while mobile medical doctors attended clinically complicated HIV patients. Because PMTCTA remained the standard of care nationally and per the guidance from the Ministry of Health, women were still allowed to opt for AZT in the PMTCTB+ study. Treatment response was monitored through routine VL testing at 6 and 12 months and annually thereafter using the Biocentric (Bandol, France) multi-manufacturer open platform for HIV-1 VL quantification on plasma samples. Stepped-up adherence counselling was performed in case of an elevated VL > 1000 copies/ml [13]. EID testing was performed using the Cobas® AmpliPrep/Cobas® TaqMan® v2.0 for the qualitative detection of HIV-1 from heel prick dried blood spots. Women missing their scheduled ART refill visits or their EID appointment at 6 weeks after birth were traced by phone as per national protocol.

### Variables and definitions

First, for HIV+ pregnant treatment naïve women, the primary outcome was time from first ANC visit to ART initiation. Censoring occurred on the date of AZT initiation, transfer out of the study facilities, death, loss to follow-up (LTFU), and latest at 6 months after the first ANC visit. Second, for women commencing ART, the primary outcome was time to occurrence of the composite unfavourable endpoint LTFU (defined as ≥4.5 months without visit) or death. Censoring occurred at the last recorded clinic visit for transfer out and latest at the end of the observation period (18 September 2015). Six-month VL test utilization was defined as the proportion of women being on ART for at least 6 months who received a VL test between 6 to 12 months after the commencement of ART. Finally, EID utilization and HIV mother to infant transmission were described. Because the date of birth was often not recorded, EID was defined as any PCR measurement done between birth and the end of the observation period. HIV transmission was described for infants who had EID test results available. For all analyses, baseline variables were measured at the time of the first ANC visit. Same-day ART initiation was defined as patients starting ART on the day of HIV diagnosis. The study enrolment period was divided into 3 phases (early: 01–06/2013, mid: 07–12/2013 and late: 01–06/2014) to account for temporal trends.

### Data management and statistical analyses

Trained data clerks entered patient information into an electronic study database. Clinical and socio-demographic data were obtained from facility-based registers and patient files. VL test results were obtained from the laboratory based electronic VL database, and birth outcomes and EID data from maternity records and child welfare registers. Baseline characteristics were described using frequency statistics and proportions. Kaplan-Meier estimates were computed and uni- and multivariate Cox proportional-hazards regression models built for time to ART initiation and treatment outcome analyses. All regression models were adjusted for baseline socio-demographic and clinical covariate information, and the final models were obtained through backwards stepwise regression and elimination of covariates with *p* > 0.15. All analyses were performed using STATA (version 12.1) [21].

## Results

### Baseline characteristics

Overall, 665 HIV-positive pregnant women attended a first ANC visit (Table 1) and 354 (53.2%) at primary care facilities. The median age was 26 years (IQR: 23, 30), the median CD4 count was 387 cells/μl (IQR: 264, 539), 506 (76.1%) had combined WHO stage I/II and 408 (61.4%) were newly diagnosed HIV+. The median gestational age was 22 weeks (IQR: 17, 26) and 131 (19.8%) were primigravida. Overall one third of patients were enrolled during each of the enrolment phases.

**Table 1:** Patient characteristics, treatment status and predictors of ART initiation in HIV+ pregnant women under PMTCTB+

Characteristics at ANC1		Treatment status				Predictors of treatment initiation			
	Eligible for ART, n (%)	Initiated ART, n (%)	Switched from AZT to ART, n (%)	AZT, n (%)	No AZT/ ART, n (%)	cHR (95% CI)	*p*	aHR (95% CI) (*n* = 665)	*p*
Total (*n* = 665)	665	496	47	70	99				
CD4									
0–349	260 (39.1)	224 (45.2)	21 (44.7)	14 (20.0)	22 (22.2)	Reference		Reference	
350–499	171 (25.7)	132 (26.6)	14 (29.8)	20 (28.6)	19 (19.2)	0.77 (0.62,0.95)	0.015	0.82 (0.66,1.02)	0.075
≥500	170 (25.6)	119 (24)	11 (23.4)	24 (34.3)	27 (27.3)	0.66 (0.53,0.83)	< 0.001	0.69 (0.56, 0.87)	0.002
Missing	64 (9.6)	21 (4.2)	1 (2.1)	12 (17.1)	31 (31.3)	0.23 (0.14,0.35)	< 0.001	0.31 (0.20,0.49)	< 0.001
WHO									
I + II	506 (76.1)	416 (83.9)	44 (93.6)	42 (60.0)	48 (48.5)	Reference		Reference	
III + IV	15 (2.3)	11 (2.2)	1 (2.1)	3 (4.3)	1 (1.0)	0.78 (0.43,1.42)	0.418	0.78 (0.43,1.42)	0.418
Missing	144 (21.7)	69 (13.9)	2 (4.3)	25 (35.7)	50 (50.5)	0.43 (0.33,0.55)	< 0.001	0.40 (0.31,0.52)	< 0.001
Agegroup (*n* = 662)									
16–24	271 (40.8)	197 (39.7)	24 (51.1)	33 (47.1)	41 (41.4)	Reference			
25–34	344 (51.7)	260 (52.4)	22 (46.8)	34 (48.6)	50 (50.5)	1.11 (0.92, 1.33)	0.284		
≥35	47 (7.1)	38 (7.7)	1 (2.1)	2 (2.9)	7 (7.1)	1.26 (0.89,1.79)	0.190		
HIV diagnosis (*n* = 658)									
New	408 (61.4)	307 (61.9)	32 (68.1)	47 (67.1)	54 (54.5)	Reference			
Previously diagnosed	250 (37.6)	185 (37.3)	15 (31.9)	22 (31.4)	43 (43.4)	1.01 (0.84,1.21)	0.920		
Trimester (*n* = 588)									
1st	64 (9.6)	50 (10.1)	2 (4.3)	7 (10.0)	7 (7.1)	Reference			
2nd	413 (62.1)	318 (64.1)	34 (72.3)	46 (65.7)	49 (49.5)	0.93 (0.69,1.25)	0.635		
3rd	111 (16.7)	82 (16.5)	6 (12.8)	10 (14.3)	19 (19.2)	0.91 (0.64,1.29)	0.599		
Pregnancy (*n* = 661)									
1st	131 (19.7)	100 (20.2)	15 (31.9)	14 (20)	17 (17.2)	Reference			
≥2nd	530 (79.7)	394 (79.4)	32 (68.1)	55 (78.6)	81 (81.8)	0.97 (0.78,1.21)	0.805		
Health care facility (*n* = 665)									
Primary	354 (53.2)	253 (51)	15 (31.9)	43 (61.4)	58 (58.6)	Reference		Reference	
Secondary	311 (46.8)	243 (49)	32 (68.1)	27 (38.6)	41 (41.4)	1.16 (0.97,1.38)	0.099	1.15(0.96,1.37)	0.132
Enrolment phase^a^									
Early	232 (34.9)	150 (30.2)	32 (68.1)	43 (61.4)	39 (39.4)	Reference		Reference	
Mid	202 (30.4)	148 (29.8)	12 (25.5)	22 (31.4)	32 (32.3)	1.37 (1.09,1.72)	0.007	1.41 (1.12,1.77)	0.003
Late	231 (34.7)	198 (39.9)	3 (6.4)	5 (7.1)	28 (28.3)	2.17 (1.75,2.70)	0.000	2.36 (1.89,2.94)	< 0.001

*cHR* crude hazard ratio, *aHR* adjusted hazard ratio

^a^Definition of study enrolment phases: early from 01/2013 to 06/2013, mid from 07/2013 to 12/2013 and late from 01/2014 to 06/2014

### ART initiation

**Fig. 1:**
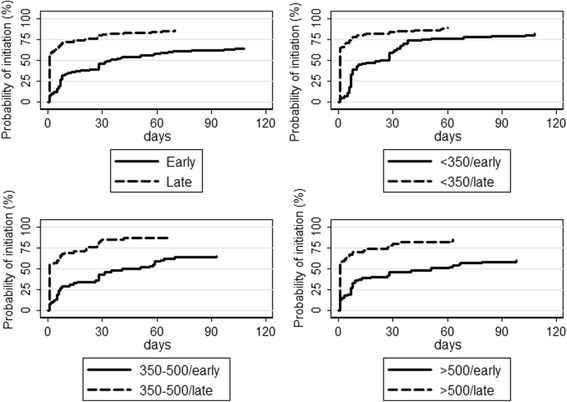
Kaplan-Meier graph of time to ART initiation stratified by CD4 group and implementation phases. *Early and late refers to the early (01/2013 to 06/2013) and late (01/2014 to 06/2014) study enrolment periods

Of 665 HIV+ pregnant women, 496 (74.6%) initiated ART (of whom 47 transitioned from single AZT therapy), 70 (10.5%) opted for AZT and 99 (14.9%) received neither. Of the 496 who initiated ART, 454 (91.5%) were on a Tenofovir+Lamivudine+Efavirenz, 3 (0.6%) were on Zidovudine+Lamivudine+Nevirapine, 3 (0.6%) were on Zidovudine+Lamivudune+Efavirenz and 36 (7.3%)) were missing ART regimen data. The proportion of women initiating ART among all individuals increased from 150 (64.7%) to 198 (85.7%) between early to late study implementation phases. Kaplan-Meier estimates for same-day ART initiation was 34.1% (95%CI: 30.7–37.9) and for three-month ART initiation was 74.4% (95%CI: 71.1-77.7). The same-day and six-month ART initiation rate increased from 8.6% (95%CI: 5.6–13.0) to 58.4% (95%CI: 52.2–64.8) and 64.2% (95%CI 58.1–70.4) to 85.7% (95%CI 80.8–89.9; *p* < 0.001) respectively between early and late enrolment phases (Fig. 1).

In multivariate analysis (Table 1), women with CD4 ≥ 500 (aHR: 0.69, 95%CI 0.56–0.87), missing CD4 (aHR: 0.31, 95% 0.2–0.49) and missing WHO staging result (aHR: 0.40, 95% 0.31–0.52) were less likely to initiate ART. The only positive predictor of ART initiation was entry into the study in later phases (mid: aHR: 1.41, 95% 1.12–1.77; late: aHR: 2.36, 95% 1.89–2.94).

### ART outcomes

Of 496 women initiated on ART, 250 (50.4%) were in care in the study clinics at the end of the observation period, 4 (0.8%) had died and 37 (7.5%) had transferred out. Of 205 (41.3%) women LTFU, 44/205 (21.5%) never returned for the first drug refill visit. Of 94/205 (45.9%) with recorded delivery date, 34 (36.2%) had the last drug refill visit before delivery. Kaplan-Meier estimated retention was 79.9% (95% CI 76–83.2), 70.7% (95% CI 66.4–74.6) and 52.9% (95% CI 47.6–57.9) at 6, 12 and 24 months. Retention between the CD4 categories was different only for the first 3 months after ART initiation (*p* = 0.01) but similar thereafter (*p* = 0.187). In the multivariate model (Table 2), ANC visit in the third trimester (aHR: 2.37, 95% CI 1.39–4.01), secondary care facility (aHR: 1.98, 95% CI 1.50–2.60) and later enrolment phases (mid aHR: 1.48, 95% CI 1.05–2.08; late aHR: 1.67, 95% CI 1.18–2.37) were associated with an increased risk of attrition while the risk was reduced for the age-group 25–34 years (aHR: 0.75, 95% 0.57–0.99). Following defaulter tracing, 34 (40.5%) of 84 mothers traced from those LTFU re-started ART after completion of the retention analysis, and their median time of treatment interruption was 5 months (IQR: 4.0, 7.3).

**Table 2:** Characteristics of women who initiated ART and stopped ART under PMTCTB+, and factors associated with all cause ART attrition

	Initiated ART, n (%)	All cause attrition, n (%)	cHR (95% CI)	p	aHR (95% CI) (*n* = 436)	p
Total	496	209				
CD4 (*n* = 496)						
0–349	224 (45.2)	80 (38.3)	Reference		Reference	
350–499	132 (26.6)	57 (27.3)	1.29 (0.92,1.82)	0.144	1.21 (0.88,1.66)	0.251
≥500	119 (24.0)	62 (29.6)	1.70 (1.22,2.37)	0.002	1.28 (0.92,1.78)	0.138
Missing	21 (4.2)	10 (4.8)	1.93 (1.00,3.73)	0.050	2.06 (0.98,4.34)	0.057
WHO (n = 496)						
I + II	416 (83.9)	176 (84.2)	Reference		Reference	
III + IV	11 (2.2)	5 (2.4)	1.24 (0.51,3.01)	0.639		
Missing	69 (13.9)	28 (13.4)	1.23 (0.82,1.84)	0.317		
Age group (*n* = 495)						
16–24	197 (39.7)	96 (45.9)	Reference		Reference	
25–34	260 (52.4)	97 (46.4)	0.70 (0.53,0.93)	0.014	0.75 (0.57,0.99)	0.046
≥35	38 (7.7)	16 (7.7)	0.86 (0.51,1.46)	0.580	0.99 (0.61,1.61)	0.979
Missing	1 (0.2)	0 (0)	___			
HIV diagnosis (*n* = 492)						
New	307 (61.9)	126 (60.3)	Reference			
Previously diagnosed	185 (37.3)	83 (39.7)	1.11 (0.84,1.47)	0.46		
Missing	4 (0.8)	0 (0)	___			
Trimester (*n* = 450)						
1st	50 (10.1)	15 (7.2)	Reference		Reference	
2nd	318 (64.1)	129 (61.7)	1.61 (0.93,2.80)	0.089	1.49 (0.94,2.38)	0.091
3rd	82 (16.5)	43 (20.6)	2.56 (1.40.4.68)	0.002	2.37 (1.39,4.01)	0.001
Missing	46 (9.3)	22 (10.5)	___			
Pregnancy (*n* = 494)						
1st	100 (21.2)	44 (21)	Reference			
≥2nd	394 (79.4)	164 (78.5)	0.87 (0.62,1.22)	0.419		
Missing	2 (0.4)	1 (0.5)	___			
Health care facility (n = 496)						
Primary	253 (51.0)	90 (43.1)	Reference		Reference	
Secondary	243 (49.0)	119 (56.9)	1.46 (1.11,1.92)	0.007	1.98 (1.50,2.60)	< 0.001
ART initiation (*n* = 496)						
Deferred	380 (76.6)	160 (76.6)	Reference			
Same-day	116 (23.4)	49 (23.4)	1.10 (0.80,1.52)	0.451		
Enrolment phase (n = 496)						
Early	150 (30.2)	63 (30.1)	Reference			
Mid	148 (29.8)	65 (31.1)	1.32 (0.93,1.88)	0.124	1.48 (1.05,2.08)	0.025
Late	198 (39.9)	81 (38.8)	1.50 (1.06,2.12)	0.024	1.67 (1.18,2.37)	0.004

*cHR* crude hazard ratio, *aHR* adjusted hazard ratio

### Viral load outcomes

Of 496 women initiated on ART, 373 were retained in care for at least 6 months, making them eligible for a first VL test. Of these 251 (67.3%) received a first VL test and 223/251 (88.8%) were virologically suppressed (< 1000 copies/mL).

### Early infant diagnosis

Of 670 infants, 359 (53.6%) received a PCR EID HIV test. A test result was available for 320 (89.1%) infants, of whom 7 (2.2%) were HIV-positive: 3/278 (1.1%) for women on ART, 2/34 (5.9%) for women on AZT and 2/31 (6.5%) for women without ART/AZT; 4/141 (2.8%) for women with CD4 < 350, 1/94 (1.1%) for CD4 350-499 and 1/85 (1.2%) for CD4 ≥ 500. For the 3 infants who tested positive with their mothers on ART, all three women had a suppressed VL test at the time of delivery and the 3 infants received the EID test more than 2 months after delivery and were exclusively breastfed.

## Discussion

The roll out of PMTCTB+ was found to be feasible in this setting. However, several challenges were encountered along the maternal treatment and infant diagnostic cascade. Overall, three quarters of eligible women initiated ART, but only half of them were retained on ART at two years. While maternal VL suppression was high and early mother to infant HIV transmission low, VL utilization and EID testing uptake was sub-optimal.

ART initiation rates were low at the beginning of the study but increased steadily over time. Several challenges encountered during the early implementation of PMTCTB+ may have played a role in this setting, such as; patients’ and health workers’ resistance to lifelong ART [20, 22, 23]. Other studies have also reported significant barriers to ART uptake for PMTCT [23–25] although this differs from the experience in Malawi where a nationwide rollout and sensitization may have improved awareness and uptake of the program [26]. As this was a pilot study, no national sensitization could be organized to promote ART acceptance specifically in women who were not eligible for lifelong ART under PMTCA [20]. In addition, AZT persisted as an option during the entire study period because PMTCTA remained the standard of care in the rest of the country. These issues were addressed in response to early poor ART uptake through repeated trainings for nurses and community health workers and through intensified community awareness and mobilization activities.

Only 53% of women were retained in care at 24 months. Attrition was highest among HIV+ women on ART at the secondary health care facility, among women who initiated ART during the last trimester of their pregnancy and for women with CD4 ≥ 500. These findings are in line with other studies [26–31]. A possible explanation is that women only initiated ART for the benefit of the unborn child, because they had limited understanding of the subsequent benefit of ART for their own health and in preventing transmission to their infant during breastfeeding [32, 33]. In addition, 9% of women receiving ART did not return for the first drug refill, similar to findings from another study in Ethiopia [31] which may reflect the importance of early treatment adherence support and retention messages especially for mothers with higher CD4 count at the start of their treatment and clinical follow-up [23].

The risk of attrition was also higher among women initiated on ART during the later enrolment phase which had a higher proportion of same-day ART initiations. Given that other observational studies have reported higher risk of attrition in same-day initiates [31, 34], we included this factor in the analysis as a possible predictor of attrition. Although no association was discovered, we still suggest that caution should be taken with same-day ART initiation as a public health approach and patient readiness should be taken into account [35].

In addition, context- and culture-specific factors probably play a major role in the attrition we observed. Many facilities are located close to the South African border. As pregnant women attending South African clinics are given incentives after delivery, it is possible that some mothers may have chosen to continue treatment there. Furthermore, in their culture many Swazi women continue to face structural barriers to ART initiation and continuation such as gender inequality, economic dependency and patriarchal social factors [36]. Women often return to their mother’s homestead for delivery and for the first few months of child rearing but move back to their own homes later. Future work could look into the role of migration in treatment discontinuation in this treatment group.

In this cohort, 67% of the women on ART received a first VL after 6–12 months; of these 89% had an undetectable VL. To our knowledge, only one other study has reported on VL outcomes of a PMTCTB+ cohort in sub-Saharan Africa and reported similar findings [37]. VL monitoring was still quite a novel practice for health workers and its routine use for treatment monitoring was still not fully established [38]. In addition, according to our data, women were often lost to follow-up before they became eligible to receive their first VL at 6 months after ART initiation. Nevertheless, the high proportion of VL suppression among those tested was encouraging and suggested good ART adherence in patients retained and receiving a VL test. This is critical if ART is to provide long term benefits to the mothers and reduce the risk of HIV transmission during breastfeeding [39–41].

EID test utilization was suboptimal at 54%. EID information was collected from child welfare registers available at the facilities. Other authors have discussed the problems with use of paper registers [42, 43]. The only way to identify infants was if their mothers’ information was included in the register, because of this, we may have missed some poorly documented EID tests. According to program managers, often infants are brought to the facility by another care giver (e.g.: grandmother), who may not have the mother’s personal details. Healthcare workers prefer not to ask caregivers (other than the biological mother) for permission to perform the HIV test on the child, in order to avoid unintentional disclosure of the mothers’ status. HIV status assessment in children is expected to improve because Swaziland has since adopted the WHO strategy to test all infants for HIV regardless of exposure at 9 months after birth [44].

Vertical mother to child transmission rates were low (2.2%) at 6 weeks (and comparable to the national estimate of 2.4% [19]) and appeared lower when the mother received ART (1.1%) when compared to AZT (5.9%) and those without AZT/ART (6.5%). Transmission rates are similar to findings from other studies [45–47]. These data, however, need to be interpreted with caution because EID results were available for only less than half of the eligible children.

This study has several limitations. First, routine paper registers and patient files were the main data source. Differences in recording practices may have led to varying completeness and quality of data which limits internal validity. This incompleteness also meant that we could not include all possible confounding factors (e.g. income, marital status and education) in our analyses limiting the robustness of our findings. Second, misclassification of the treatment outcome was possible. Although national standard operating procedures required a routine phone call three days after a missed appointment in order to re-engage women into care or ascertain the outcome, 60% of women could not be contacted due to incorrect contact details (phone numbers did not exist, were unreachable, or somebody else answered the phone). Weak ascertainment of ART outcomes and suboptimal implementation of physical defaulter tracing activities may have inflated attrition rates [48, 49]. Third, this observational study was likely affected by temporal trends such as patient and community mobilization as well as countrywide adoption and scale-up of PMTCTB+ in the second half of 2014. We addressed this by dividing the study implementation period into 3 phases. External validity is a strength of this study. PMTCTB+ was implemented under routine conditions in predominantly rural government clinics. Therefore, limitations faced in this high HIV prevalence setting likely apply to many other contexts in southern Africa.

## Conclusions

Accelerated access to ART for pregnant women (PMTCTB+) was feasible within a pilot implementation project under routine public health service. While ART initiation rates increased over time, high rates of ART attrition emerged as a programmatic challenge. Documented maternal VL suppression was high and vertical HIV transmission low, however, the uptake for VL and EID testing was suboptimal. This, together with high attrition makes judgement of the magnitude of the public health impact uncertain. Specific aspects in PMTCTB+ programming need further strengthening, in particular, paying attention to community sensitisation and training of staff, as well as to the continuity of care and follow-up for both the mothers and their infants.
